# Induction of high tolerance to artemisinin by sub-lethal administration: A new *in vitro* model of P. *falciparum*

**DOI:** 10.1371/journal.pone.0191084

**Published:** 2018-01-17

**Authors:** Serena De Lucia, Ioannis Tsamesidis, Maria Carmina Pau, Kristina R. Kesely, Antonella Pantaleo, Francesco Turrini

**Affiliations:** 1 Department of Oncology, University of Turin, Turin, Italy; 2 Department of Medicine, Section of Internal Medicine, University of Verona, Verona, Italy; 3 Department of Biomedical Sciences, University of Sassari, Sassari, Italy; 4 Department of Biochemistry, Purdue University, West Lafayette, United States of America; Université Pierre et Marie Curie, FRANCE

## Abstract

Artemisinin resistance is a major threat to malaria control efforts. Resistance is characterized by an increase in the Plasmodium *falciparum* parasite clearance half-life following treatment with artemisinin-based combination therapies (ACTs) and an increase in the percentage of surviving parasites. The remarkably short blood half-life of artemisinin derivatives may contribute to drug-resistance, possibly through factors including sub-lethal plasma concentrations and inadequate exposure. Here we selected for a new strain of artemisinin resistant parasites, termed the artemisinin resistant strain 1 (ARS1), by treating P. *falciparum* Palo Alto (PA) cultures with sub-lethal concentrations of dihydroartemisinin (DHA). The resistance phenotype was maintained for over 1 year through monthly maintenance treatments with low doses of 2.5 nM DHA. There was a moderate increase in the DHA IC_50_ in ARS1 when compared with parental strain PA after 72 h of drug exposure (from 0.68 nM to 2 nM DHA). In addition, ARS1 survived treatment physiologically relevant DHA concentrations (700 nM) observed in patients. Furthermore, we confirmed a lack of cross-resistance against a panel of antimalarials commonly used as partner drugs in ACTs. Finally, ARS1 did not contain P*fk13* propeller domain mutations associated with ART resistance in the Greater Mekong Region. With a stable growth rate, ARS1 represents a valuable tool for the development of new antimalarial compounds and studies to further elucidate the mechanisms of ART resistance.

## Introduction

Plasmodium *falciparum* is the most lethal of the malaria known to infect humans [1], and was responsible for nearly 212 million new cases and 429.000 deaths in 2016 [2]. Artemisinin derivatives (ART) represent the most widely used anti-malarial drugs. Derived from the plant *Artemisia annua*, ART contain an endoperoxide ring essential for its potent and rapid antimalarial activity [3, 4]. Although ring-stage parasites are most sensitive to drug treatment, ART are active against all asexual stages of parasite development [5]. Unfortunately, the use of ART as mono-therapies has contributed to the increase and spread of artemisinin resistant parasites, and remains a major threat for malaria control efforts in South East Asia [6]. Although artemisinin derivatives are now only used in combination with slow-acting partner drugs as ACTs to deter drug—resistance, however, resistance to partner drugs has been detected [7, 8], further necessitating the development of new and effective antimalarials [9, 10].

Artemisinin resistance is defined by a delay in parasite clearance following 3 days (DPC3) of treatment with ACTs, but, many times, parasites become resistant to the partner drug in addition to ART [11]. Partner drug resistance is characterized by recrudescence of the infection after 28 or even 42 days following ACT treatment [12]. For this reason, malaria parasite are evaluated 3 days and 28 days following treatment for a lack of adequate clinical and parasitological response at day 28 (ACPR28) [13, 14, 15, 16]. ART rely on their rapid conversion to reactive free radicals for potent antimalarial activity. However, their very short clearance half-life (~1h) combined with under dosage of infected patients and incomplete drug treatment may contribute to the occurrence and spread of ART resistance [17, 18, 19]. Exacerbating the situation, patients frequently undergo incomplete treatment [20, 21]. ART resistant parasites may have acquired the ability to survive under the sub-lethal drug concentrations or insufficient exposure to artemisinin, similar to that seen in bacterial resistance to antimicrobial agents [22, 23, 24].

Stable ART resistant strains of P. *falciparum*, have been obtained from infected patients while others have been manipulated to induce artemisinin resistance by subjecting parasites to increasing concentrations of DHA *in vitro* [22, 25, 26, 27].

The heterogeneity and number of factors that can contribute to ART resistance have been evidenced by the amount of studies performed in a similar manner *in vitro* [28, 29]. Interestingly, the over-expression of P. *falciparum* malaria genes related to cell repair mechanisms have been identified and not only support research demonstrating ART generate free radicals for antimalarial activity, but also the rapid response of parasites to increasing drug pressure by inducing a series of protective and repair mechanisms [30, 31].

Laboratory-adapted strains of P. *falciparum* contain phenotypes uncharacteristic to those found in clinical isolates, or parasite resistance found in patients [32]. While all these strains may be ART resistant, not all artemisinin resistance phenotypes are the same. In addition, the type manipulation used to induce artemisinin resistance *in vitro* can induce a similar uncharacteristic phenotype. For example, a large number of dormant parasites are induced by short treatments with high DHA concentrations, but evidence of similarly dormant parasites during infection is lacking [33].

Finally, it has been reported that mutations in the P. *falciparum* kelch-13 propeller (P*fk13*) gene are associated with ART resistant malaria in the Greater Mekong Sub-region and in China-Myanmar [34, 35, 36]. It has been suggested this resistance is due to the dysregulation of Phosphatidylinositol 3-phospate metabolism, which is critical for parasite survival [37, 38, 39]. Although ACTs have been widely used for over a decade, no P*fk13* propeller mutations associated with ART resistance have been reported for in African P. *falciparum* populations [40, 41]. These results strongly support the involvement of multiple factors with delayed parasite clearance after ACT treatment and the importance of preventing parasite tolerance to the partner drug.

With these findings in mind, we set out to select an artemisinin resistant strain by exposing parasites to sub-lethal DHA concentrations and then progressively shortening the time intervals between drug treatments. Cultures were monitored, taking great care to prevent the accumulation of dormant parasites. We now present a novel P. *falciparum* artemisinin resistant strain (ARS1) that possess a high tolerance against sub-lethal DHA concentrations and this stable strain will help elucidate ART resistance mechanisms and direct efforts to test new antimalarial compounds.

## Materials and methods

### Plasmodium *falciparum* cultures

Three P. *falciparum* parasites strains were used in this study and obtained from field isolate. The Palo Alto (PA) strain was isolated from a Ugandan patient and is considered as a reference strain due to its high genetic stability. PA has historically been used as a strain to study drug susceptibility in P. *falciparum in vitro* [42]. The FCR3 strain derived from Gambian parasites and is genetically related with PA and it has been used to generate a chloroquine (CQ) and pyrimethamine (PM) resistant strain [22, 42]. The HB3A parasites strain derived from Honduras and has been used to induce chloroquine resistance [43, 22].

### Plasmodium *falciparum*-infected red blood cell (I-RBC) culture maintenance

P. *falciparum* strains PA, FCR3, HB3A and the ARS1 strain, selected in this study (mycoplasma free), were maintained in culture with fresh red blood cells RBCs (Rh+) from healthy volunteers of both sexes. Human blood samples were used only to sustain the parasites *in vitro* cultures. Blood samples used to perform the present study were obtained from Banca del sangue C/O Ospedale Molinette, Torino with written informed consent. This study was conducted in accordance with Good Clinical Practice guidelines and the Declaration of Helsinki. No ethical approval has been requested. RBCs were separated from plasma and leukocytes by three washes in RPMI 1640 medium containing 25 mM HEPES (R5886 Sigma Aldrich), and then resuspended in RPMI 1640 + HEPES medium supplemented with 200 mM L-glutamine (59202C Sigma Aldrich), 2 mM glucose and gentamicin (80 mg/mL). For complete growth medium, this was further supplemented with 1% SAG-M solution, (150 mM NaCl, 1.25 mM adenine, 30 mM glucose, 145 mM mannitol) and heat-inactivated human serum. I-RBC cultures were maintained at 2–5% parasitemia (1% haematocrit) at 37°C in an air/CO_2_-athmosphere of 95/5% (vol/vol). All assays were performed at this parasitemia and hematocrit unless otherwise described.

Cultures were synchronized weekly by Percoll separation [44] or 5% sorbitol solution treatment [45]. The synchronization process did not influence the generation or phenotype of the artemisinin resistant strain. Parasite viability and parasitemia were monitored by Diff-Quick stained thin blood smears and light microscopy (Carl Zeiss Standard Microscope Lamphouse 467230). Parasitemia was defined as the number of parasites/number of RBCs counted, for a total of 5000 RBCs. Two thin smears per condition were counted 3 separate times by each of three operators.

### Selection of artemisinin resistant strain 1 (ARS1) following DHA treatment

DHA (D7439 Sigma-Aldrich) was suspended in sterile dimethyl sulfoxide (DMSO) and subsequently diluted in RPMI 1640 + HEPES prior to I-RBC treatment.

For the first week (D1 –D7): i) synchronous cultures of PA, FCR3 and HB3A were supplemented daily with three concentrations of DHA (1.25, 2.5 and 5 nM) along with an untreated control. For cultures with a percentage of viable parasites lower than 1%, the daily treatments were stopped and only re-applied with a parasitemia of greater than 2.5%. ii) Before every new treatment, I-RBC cultures were washed once in RPMI 1640 + HEPES and resuspended in fresh growth medium containing the same concentration of DHA.

**Fig 1:**
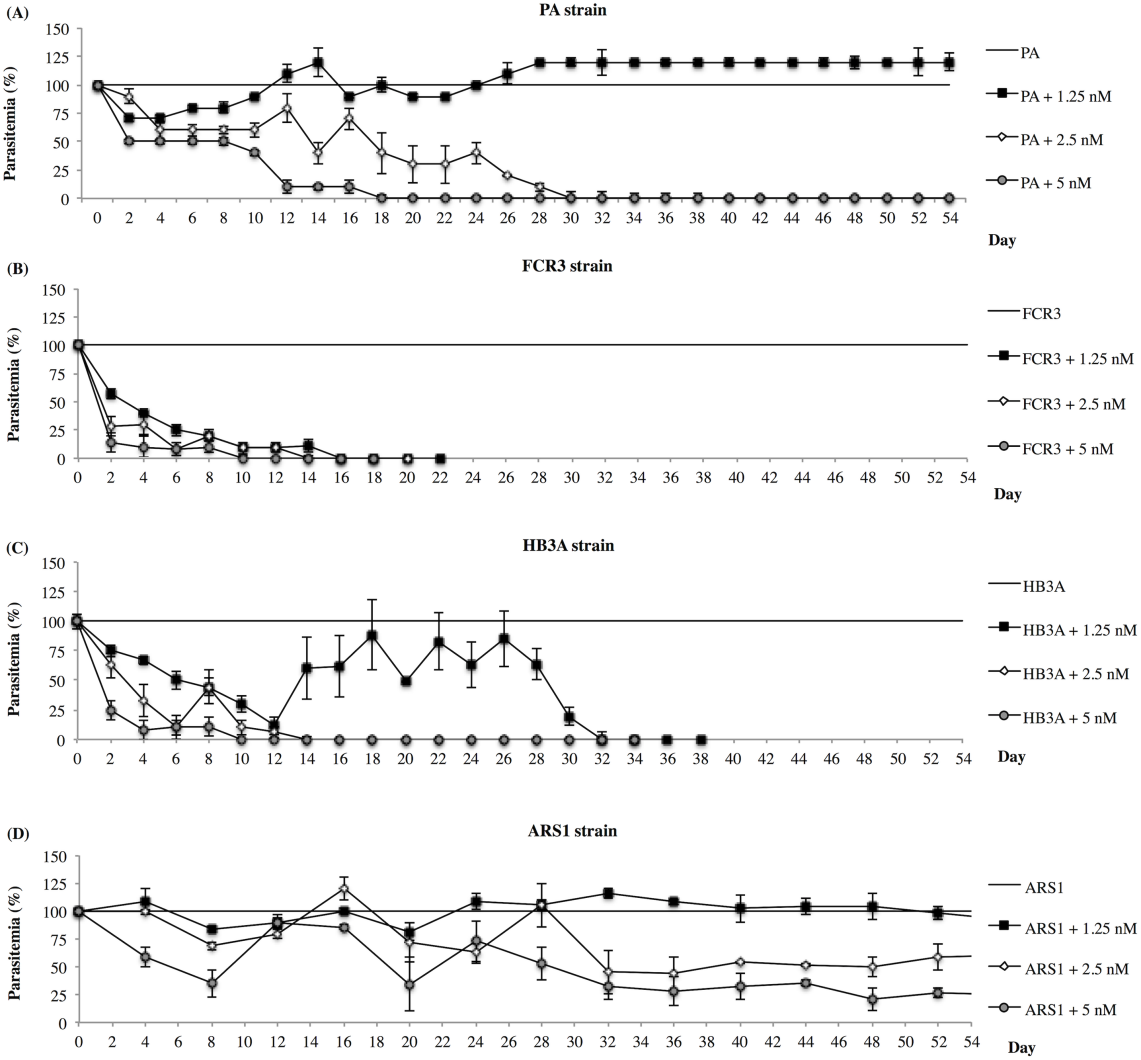
Response of P. *falciparum* cultures PA, FCR3, HB3A and ARS1 to DHA. PA (A), FCR3 (B), HB3A (C) and ARS1 (D) cultures were first treated daily with 1.25, 2.5 and 5 nM for 1 week and then treated weekly for 7 weeks, for a total of 54 days. Mature and immature parasites were counted. Data were normalized the parasitemia of untreated cultures determined by light microscopy of Giemsa-stained smears. Mean +/- SD of 5 independent experiments is shown.

For the next 7 weeks (D8 –D54): i) PA, FCR3 and HB3A were synchronized with 80% Percoll every 7–8 days, ii) treated with their respective concentration of DHA once a week (the day following Percoll synchronization), iii) the growth medium was changed every 24 h. Parasitemia was determined every day from the start of the study. When cultures were completely devoid of viable parasites for at least 7 days (analysing two thin smears every day), the cultures were considered parasite free. Fig 1 demonstrates parasite morphology from D0 to D54 of the parental PA strain and the surviving ARS1 strain with the average parasitemia reported as previously described with two operators and as a percent of the untreated control. To maintain the ARS1 strain selected from PA: i) ARS1 cultures were synchronized every 7 days with 80% Percoll to isolate trophozoite stage parasites and treated with Sorbitol 5% 24 h later to isolate ring stage parasites; ii) cultures were treated with 2.5 nM of DHA monthly at ring stage 24 h following sorbitol synchronization; iii) parasite cultures were incubated with DHA for 48 h and then replaced with fresh growth medium which was changed every 48 h; iv) this monthly DHA treatment was continued for over 1-year period.

### Analysis of long-term treatment of strains PA and ARS1 with repeated DHA treatments

**Fig 2:**
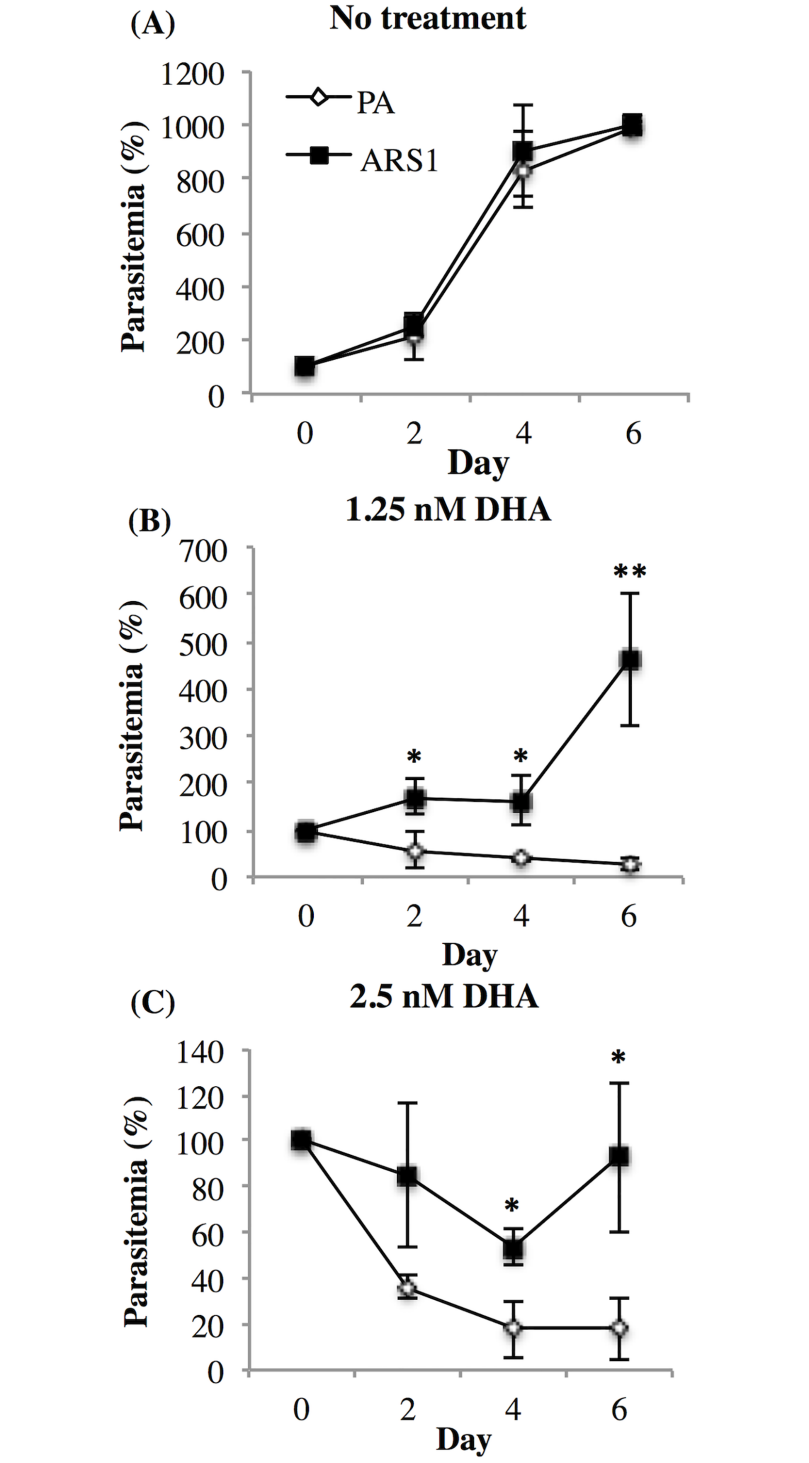
Survival of ARS1 to increased frequency of DHA treatments every 48 h. PA and ARS1 were analysed without treatment (A) and after repeated treatments every 48 h with 1.25 nM (B) or 2.5 nM (C) DHA, by counting mature and immature parasites. Data were normalized to the control parasitemia and analysed by light microscopy of Giemsa-stained thin smears. The medium was changed every 48 h. Mean +/- SD of 5 independent experiments is shown. Significant differences between strains are indicated by (*) at p < 0,05 and (**) at p < 0,01.

PA and ARS1 were treated with 1.25 and 2.5 nM DHA, respectively every 48 h for a total of 144 h in order to evaluate the PA and ARS1 behaviour assessed with different frequency DHA pressure (Fig 2) or with 2 nM DHA every 12 or 24 h for a 72 h. Parasitemia was determined as previously described.

### Analysis of DHA’s effect on parasite maturation and death

PA and ARS1 ring stage cultures were treated with 0.6, 1.25, 2.5, 5 and 10 nM of DHA and evaluated at 24 and 48 h following drug addition. The effect of DHA treatment on P. *falciparum* maturation and death was evaluated by determining the parasitemia of each maturation stages: ring, trophozoite and schizont, and for intracellular dead parasites (pyknotic) in both DHA-treated and untreated control cultures. The percentage of each stage of live parasite and the number of dead parasites present in DHA treated cultures were expressed as the % parasitemia relative to the untreated cultures.

### Rings-stage Survival Assay (RSA^0-3h^)

The *in* vitro RSA^0-3h^ assay was performed using the method described by Worldwide Antimalarial Resistance Network (WWARN) (2013) and by Witkowski et al. to demonstrate the presence of reduced sensitivity to DHA in the newly selected ARS1 strain [46, 47, 48].

Briefly, the 0–3 h post invasion, ring stage parasites were exposed to 700 nM DHA for 6 h. The cultures were washed to remove the drug and then cultured with the complete medium for additional 66 h. The proportion of viable parasites was counted in the second generation parasites with normal morphology.

As described by the WHO guidelines, we calculated the survival rate (%) and the growth rate: i) the percentage of survival rate was defined as the DHA-exposed parasitemia at 72 h/non-exposed parasitemia at 72 h x 100; ii) the growth rate was defined as the non-exposed parasitemia at 72 h/the initial parasitemia at 0 h. Every thin smear were counted by microscopy via Giemsa stained evaluating a total of 10000 RBCs. Two thin smears per condition were counted 3 separate times by each of three operators. Values obtained for % of survival rate of DHA-treated are interpretable if they correspond to a growth rate ≥ 1.5%.

### IC_50_ of P. *falciparum* strains treated with antimalarial drugs

Synchronous ring stage cultures of strains PA, ARS1, FCR3 and HB3A were treated with DHA, mefloquine (MQ), piperaquine (PPQ), amodiaquine (AQ) and lumefantrine (LMF) and parasitemia checked every 24 h following drug treatment. The scalar concentrations of each drug included 0.6, 1.25, 2.5, 5,10, 20 and 40 nM for DHA and 5, 10, 25, 50, 75, 100 and 200 nM for AQ, MQ, PPQ and LMF. To determine the IC_50_ of each drug, cultures were treated with a single-pulse of drug and incubated for 48 h [49, 28, 50]. IC_50_ values were determined using ICestimator software by schizont counting (http://www.antimalarial-icestimator.net/runregression1.2.htm) [51]. The half-life was considered relevant when the R^2^ value of the slope regression line was higher than 0.8.

### Treatments with DHA and MQ combined

PA and ARS1 cultures were treated with DHA alone or in combination with MQ to determine if there is synergism between the two drugs and whether ARS1 is sensitive to this type of ACT. Cultures were synchronized using Percoll/5% sorbitol treatment and assay initiated at a 5% parasitemia and 1% haematocrit. IC_50_ values determined at 24, 48 and 72 h following drug treatment: DHA (0.6, 1.25, 2.5, 5, 10, 20 and 40 nM) +/- supplemented with 5 or 10 nM MQ where described.

### P. *falciparum* DNA extraction

A salting out method was used to extract parasite DNA from 100μL of I-RBCs according to standard procedures by Miller et al. [52]. Briefly, RBCs were lysed with Red Cells Lysis Buffer (RCLB) solution (Tris-HCl 10 mM, MgCl_2_ 5 mM and NaCl 10 mM, pH 7.6) and the cell pellet isolated and incubated at 55°C for 20’with White Cells Lysis Buffer (WCLB) solution (Tris-HCl 10 mM, EDTA 10 mM and NaCl 50 mM, pH 7.6) in the presence of SDS (10%) and proteinase K (20 mg/ml). The addition of a saturated salt solution (NaCl 6M) purified the DNA, which was then precipitated following the addition of isopropanol. After a 70% ethanol wash, the final DNA pellet was re-suspended in sterile H_2_O and analyzed for purity by determining the ratio of absorbance at 260 and 280 nm.

### Nested-PCR analysis of strains PA and ARS1 cultures

#### Sequencing the P*fk13* propeller domain

The P. *falciparum* k13 propeller domain gene was evaluated for any mutations by using nested PCR to amplify the gene previously described by Ariey et al. [32]. Amplified PCR products were purified using Exo I & Fact AP (Carlo Erba) and sequenced by Macrogen, Inc. (Netherlands). Electroferograms were visualized and analyzed with ApoE software, and alignments were performed with Muscle 3.8 software using the k13 sequence of the 3D7 clone (PF3D7_1343700) as the reference standard.

#### Genotyping of *msp1*, *msp2* and *glurp* with nested-PCR in PA and ARS1 strain

The genetic characteristics of P. *falciparum* polymorphic genes, such as the Merozoite Surface proteins 1 and 2 (*msp1 and msp2*) and the Glutamate-rich protein (*glurp*) are commonly assessed in malaria endemic areas to discriminate recrudescence from re-infecting parasites alleles. Nested PCR was used to amplify the polymorphic regions of *msp1*, *msp2* and *glurp* using family-specific primers, previously described by Snounou [53].

Briefly, in the primary reaction, oligonucleotide primers corresponded to conserved sequences within *msp1* (block 2), *msp2* (block 3) and *glurp* in the primary reaction. In the nested reaction, separate primer pairs were used that targeted the respective allelic types of *msp1* (K1, MAD20, and RO33) *msp2* (FC27 and IC3D7) and *glurp* were used [54]. The PCR products were separated on 1.5% agarose and visualized under UV illumination.

### Statistical analysis

Error bars indicate the average ± SD. The statistical significance is estimated with two-tailed Student’s t-test performed with Microsoft Excel software.

## Results

### Selection for a new artemisinin resistant strain

We first performed experiments to select for artemisinin resistant parasites by experimentally determining the lowest concentration of DHA that would enable continuous culture of P. *falciparum* strains PA, FCR3 and H3BA when subjected to intermittent sub-lethal drug exposure. The IC_50_ values (Table 1) among the three strains revealed slight variations in sensitivity to DHA treatment. The IC_50_ values of DHA in HB3A after 24 and 48 h, 2.14 and 2.27 nM, respectively, were higher than PA and FCR3 and supported by Ding et al. and Cui et al. [28, 29]. Variations in DHA sensitivity among the malaria strains can be attributed to the nature of each individual strain such as their origin, laboratory adaptation, and length of propagation [22]. In addition we obtained IC_50_ values at 24 h in addition to the 48 h and 72 h due to the characteristic short half-life of DHA in patients and in cultures and to evaluate its final antimalarial efficacy [55]. These IC_50_ values provided a baseline that was used to evaluate parasite sensitivity to DHA during the selection process.

**Table 1:** IC_50_ values of DHA, MQ, PPQ, AQ, LMF treated cultures of P. *falciparum* strains PA, ARS1, FCR3 and HB3A.

Strain of P. *falciparum*	Drug *in vitro* tested	IC_50_ ± SD (24 h)	IC_50_ ± SD (48 h)	IC_50_ ± SD (72 h)
PA	DHA	2.21 ± 0.4 nM	0.94 ± 0.1 nM	0.68 ± 0.2 nM
	MQ	19.75 ± 0.3 nM	6.30 ± 0.1 nM	5.49 ± 0.92 nM
	PPQ	20.93 ± 1.4 nM	19.19 ± 1.9 nM	16.20 ± 1.1 nM
	AQ	21.03 ± 1.4 nM	15.80 ± 1.8 nM	14.05 ± 2.1 nM
	LMF	22.64 ± 0.2 nM	18.50 ± 2.4 nM	17.12 ± 0.8 nM
ARS1	DHA	3.17 ± 0.5 nM	2.5 ± 0.2 nM	2.0 ± 0.3 nM
	MQ	18.66 ± 1.8 nM	5.54 ± 0.7 nM	4.78 ± 0.6 nM
	PPQ	19.90 ± 1.7 nM	11.66 ± 0.8 nM	8.65 ± 0.3 nM
	AQ	19.46 ± 0.6 nM	14.45 ± 1.4 nM	14.2 ± 0.8 nM
	LMF	21.29 ± 1.3 nM	17.55 ± 2.4 nM	16.99 ± 1.52 nM
FCR3	DHA	1.73 ± 0.6 nM	1.60 ± 0.3 nM	N/D
HB3A	DHA	2.14 ± 0.3 nM	2.27 ± 0.05 nM	N/D

DHA, dihydroartemisinin; MQ, mefloquine; PPQ, piperaquine; AQ, amodiaquine; LMF, lumefantrine.

All experiments were performed at least five times independently. Values are expressed in percentage of viable parasites ± SD.

For the selection process, parasite cultures were exposed to 1.25, 2.5 and 5 nM DHA every 24 h for 1 week, then washed and resuspended in drugless medium for at most 7 weeks, which included a single exposure for 24 h every week (see Materials and methods). Over time, PA cultures gained the ability to grow at a consistent rate during the weekly treatments with 1.25 nM DHA (Fig 1A). We never observed the same drug-adaptation with FCR3 or HB3A (Fig 1B and 1C). A slight growth recovery was observed during week 2 in HB3A but parasitemia declined sharply and flat lined by day 12 (Fig 1C). The higher DHA concentrations (2.5 and 5 nM) were toxic to the parasites and failed to select for any drug resistant parasites. We monitored the parasitemia of PA, FCR3, and HB3A so long as: i) there were viable parasites growing in culture and ii) the growth rate was consistent or increased over a period time. The evaluation of FCR3 and HB3A were terminated prematurely due to the complete lack of viable parasites 22 and 38 days following DHA treatment, respectively. PA was monitored for the duration of the 8-week study. After daily application of DHA during the first week, PA cultures alone contained viable parasites and the cultures appeared to be desensitized to DHA and, thus, were renamed to artemisinin resistant strain 1 (ARS1). ARS1 was evaluated for a total of 54 days after which the growth rate stabilized (the stabilization of the culture started on 32^nd^ day) and parasites consistently displayed low susceptibility to the same DHA concentration used during the selection period. At 5 nM, ARS1 was still capable of maintaining a survival rate of 20%, corroborating our initial findings. It appeared that the actual selection for ARS1 was made after the first week of daily DHA treatments and the following 7 weeks of weekly 1.25 and 2.5 nM DHA treatments served to maintain this new strain with decreased sensitivity to DHA (Fig 1D). Similarly, a 40% increase in IC_50_ was observed in ARS1 when compared with values from parental strain PA, 48 h following exposure to DHA (Table 1).

A key response of resistant parasite populations to sub-lethal drug concentrations was to recover following treatment, evidenced by an increase in parasitemia. Recovery times are specific to each strain used to the antimalarial mechanism, and so, we reduced the time interval between DHA treatments from weekly to every 48 h (Fig 2). Growth curves of ARS1 and PA are comparable in the absence of DHA (Fig 2A). Following the addition of 1.25 nM (Fig 2B) and 2.5 nM (Fig 2C) DHA treated every 48 h, only ARS1 parasitemia remained increased by 400% and 80%, respectively, from the initial value. On the other hand, PA cultures did not survive similar DHA treatments and parasitemia progressively decreased. These findings suggest that damage inflicted on ARS1 is reversible since the parasites were able to proliferate under these DHA concentrations.

**Fig 3:**
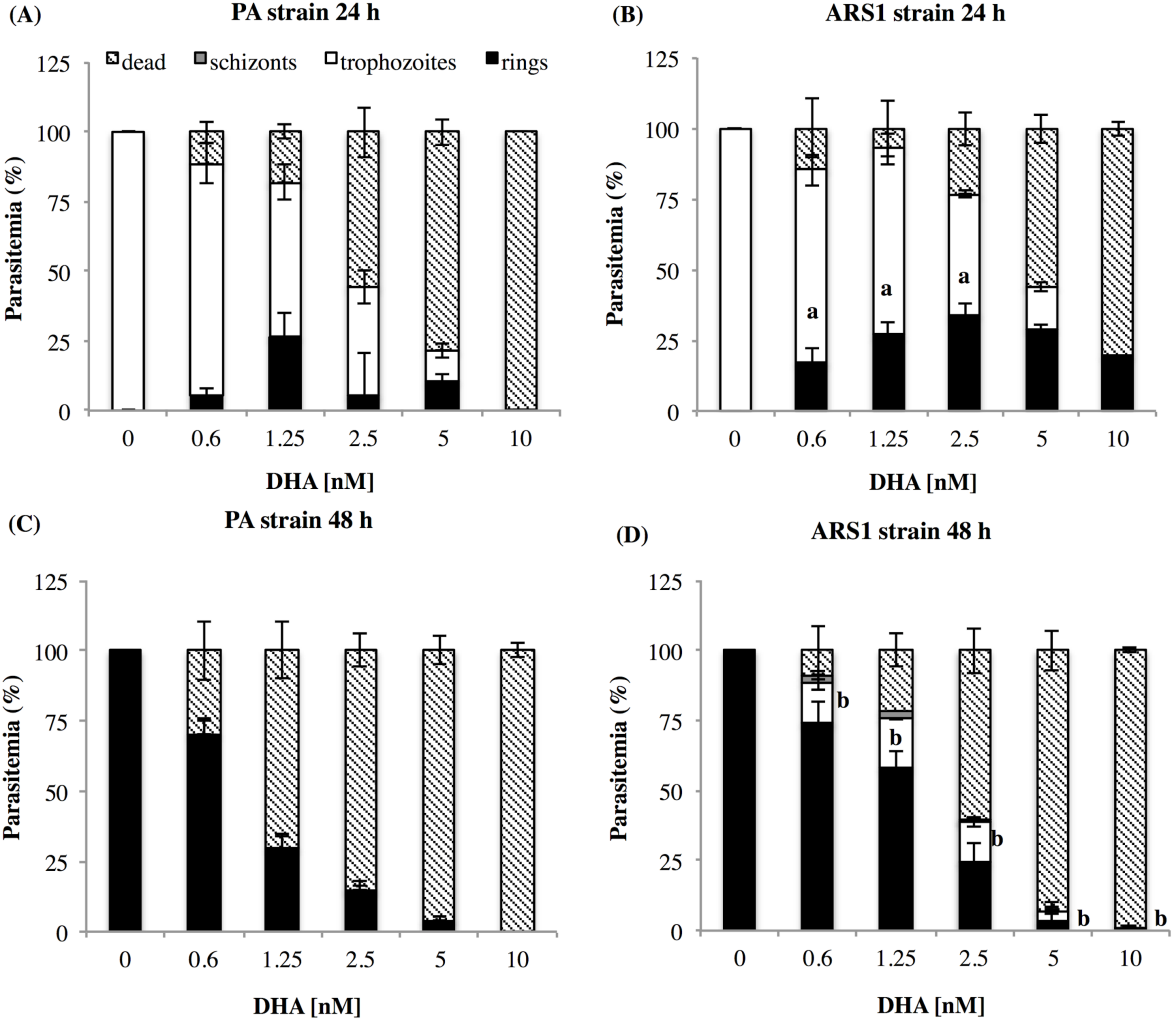
Effect of PA and ARS1 on parasite maturation following DHA treatment. The percentages of rings, trophozoites, schizonts and dead parasites were determined after treatment of ring stage parasitized cultures of the PA (A, C) and ARS1 (B, D) strains exposed to 0.6, 1.25, 2.5, 5 and 10 nM of DHA for 48 h. Parasitemia was measured at both 24 h (A) (B) and 48 h (C) (D) after treatment via light microscopy of Giemsa-stained thin smears. Data were normalized to the untreated control parasitemia. The differences of immature parasites stage of ARS1 compared to PA are indicated by: (a) p < 0.05 at 24 h, (b) p < 0.05 at 48 h.

While monitoring parasite recovery during the previous DHA treatments, the stage synchronicity of ARS1 decreased compared to PA. To better understand the effect of DHA on parasite maturation and death, ring-stage cultures were exposed to increasing concentrations for 48 h (Fig 3). As indicated by the relative percentage of rings, trophozoites, schizonts and dead parasites, a high number of parasites survived in ARS1 at concentrations up to 10 nM by 24 h, but decreased to levels observed in PA by 48 h. A significant amount of ARS1 parasites survived at lower DHA concentrations compared with PA. Untreated cultures were at the expected ring stage. The presence of trophozoites and schizonts in ARS1 signified parasite maturation was negatively affected and slower than untreated cultures. Overall, the percentage of immature parasites was higher in ARS1 than in PA at both 24 h (Fig 3A and 3B) and 48 h (Fig 3C and 3D). It’s possible that the DHA treated ARS1 parasites entered a state of quiescence at the ring stage or their development is delayed after 24 h which could confer the artemisinin tolerance.

This set of experiments demonstrates the ability to select for an artemisinin resistant strain from an initially sensitive to DHA by using sub-lethal concentrations. The results suggest that resistant parasites may be selected for during the first division cycles that follow sub-lethal damage exerted by DHA.

### Characterization of the newly generated ARS1 strain

**Fig 4:**
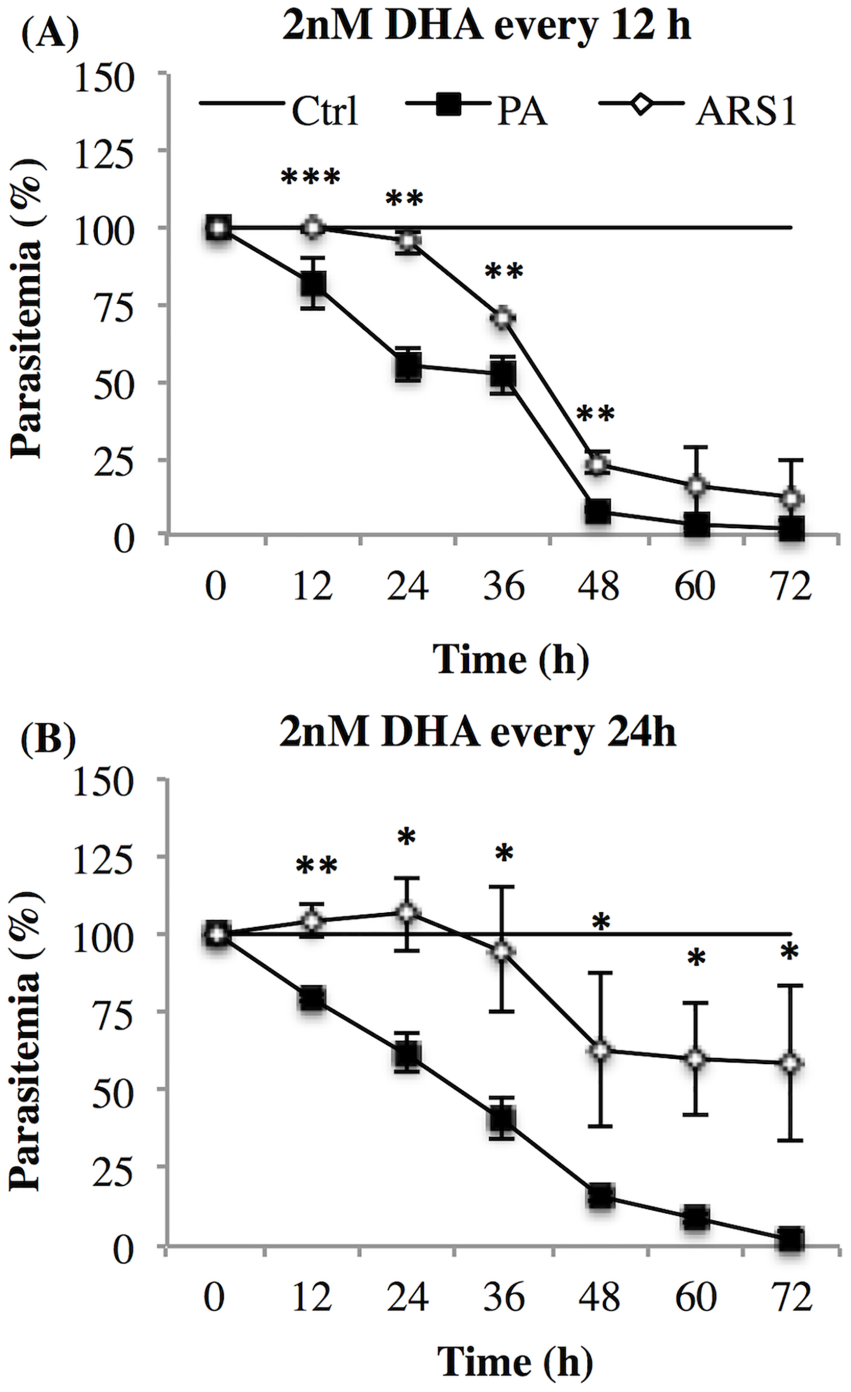
Effect of constant DHA pressure on PA and ARS1 cultures. PA and ARS1 were treated every 12 h (A) and 24 h (B) for up to 72 h, with 2 nM of DHA. Parasitemia was determined by microscopy of Giemsa-stained thin smears and normalized on the control parasitemia. Control parasitemia was determined from untreated PA and ARS1 strains maintained under the same conditions as the DHA treated. Means +/- SD of 3 independent experiments is shown. Significant differences of parasitemia between strains are indicated by (*) at p < 0.05, (**) at p < 0.01 and (***) at p < 0.001.

We next sought to evaluate ARS1 resilience to continuous drug pressure, so we replenished cultures with 2 nM DHA every 12 or 24 h. Replacement every 12 h was nearly lethal to ARS1, which appeared to require a longer period of time for parasite recovery between drug treatments. The decrease in parasitemia of ARS1 paralleled that of PA (Fig 4A). Replacement of DHA every 24 h reflects drug treatment schedule in patients. ARS1 survived this type of treatment, while it was lethal to PA, killing nearly 100% of parasites after 3 days of treatment (Fig 4B). On the contrary, ARS1 had a growth rate similar to that of untreated ARS1 cultures.

To maintain a drug resistance phenotype *in vitro*, it is critical to know the maximum time interval that can be allowed between drug treatments. This helps to evaluate a strain’s stability of drug resistance. Over 1-year period, we treated ARS1 with DHA concentrations up to 20 nM to determine the minimum concentration and maximum period of time ARS1 parasites maintain the artemisinin resistant phenotype.

After 2–3 months without DHA pressure, ARS1 lost its resistant phenotype. Parasites continue to carry the previously characterized phenotype with at least 1.25 nM DHA administered monthly. It was under monthly treatments with 2.5 nM DHA that the resistance phenotype was conserved for over 1 year. Repeated freezing and thawing of cultures using the standard protocols that employ high concentrations of glycerol did not affect this resistance either.

The Rings-stage Survival Assay (RSA^0-3h^) is a common assay to assess a parasite tolerance to DHA or other antimalarial drug. Briefly, parasite cultures were exposed to a single pulse of 700 nM DHA for 6 h using young ring-stage parasites 0-3h post-invasion after culture adaptation. This type of treatment exemplifies DHA’s short half-life in patients in addition to assessing parasite tolerance to a physiologically relevant dose of DHA. Supporting previous results, ARS1 possessed a higher tolerance to 700 nM DHA than PA, where a remarkable drop in parasitemia was observed. Results of this assay are represented by the survival rate (%) of PA and ARS1 that correspond to 0.51% and 6.8%, respectively. The percent survival rate is interpretable if the growth rate is ≥ 1.5 (see Materials and methods). In fact, the growth rate of PA and ARS1 was 8.2 ± 4.1 and 4.7 ± 0.8, respectively (Table 2).

**Table 2:** Growth rate and survival rate (%) in *in vitro* RSA^0-3h^ with 700 nM DHA at 72 h after ARS1 selection.

Strain of P. *falciparum*	Growth rate	Survival rate (%)	*p-*value vs PA
PA	8.2 ± 4.1	0.51 ± 0.46	**/**
ARS1	4.7 ± 0.8	6.8 ± 0.58	0.011

Values of growth rates are expressed as non-exposed parasitemia at 72 h/initial parasitemia ± SD; values of survival rates are expressed as (DHA-exposed parasitemia at 72 h/non-exposed parasitemia 72 h) x 100 ± SD. All experiments were performed four times independently.

In this assay, the resistance to treatment appears not due to its ability to enter a dormant or quiescent state but to the ability of ARS1 to recover and overcome the effects of DHA treatment (S1 Fig).

This set of experiments show that ARS1 not only possesses quite stable characteristics in terms of IC_50_, but, even more important, the marked capability of ARS1 strain survival at a constant rate with DHA concentrations that kill 100% of parental PA strain parasites following 3 days of treatment. In addition, ARS1 survived under the RSA^0-3h^ conditions mimicking those present in plasma of drug-treated patients.

### Sensitivity of ARS1 to treatment with other antimalarials

ART are no longer used as mono-therapies to treat malaria infections and are now only prescribed in combination with a partner drugs as ACTs. Partner antimalarials are complimentary to ART and serve to compensate for the short half-lives of ARTs and improve parasite clearance by counteracting the selection of resistant parasites.

Unfortunately, artemisinin resistance has been observed in patients treated with ACTs and for this reason, it was necessary to assess ARS1 for multidrug resistance to commonly used anti-malarial drugs with longer half-lives. IC_50_ values were determined at 24, 48 and 72 h following treatment to evaluate parasite response MQ, AMQ, LMF and PPQ (Table 1). The IC_50_ concentrations of these four drugs were similar in both ARS1 and PA and parasites susceptible to each antimalarial.

Cultures were then treated with DHA and MQ, a combination commonly used in ACTs where the long half-life of MQ compensates for the fast acting but short lived DHA.

**Fig 5:**
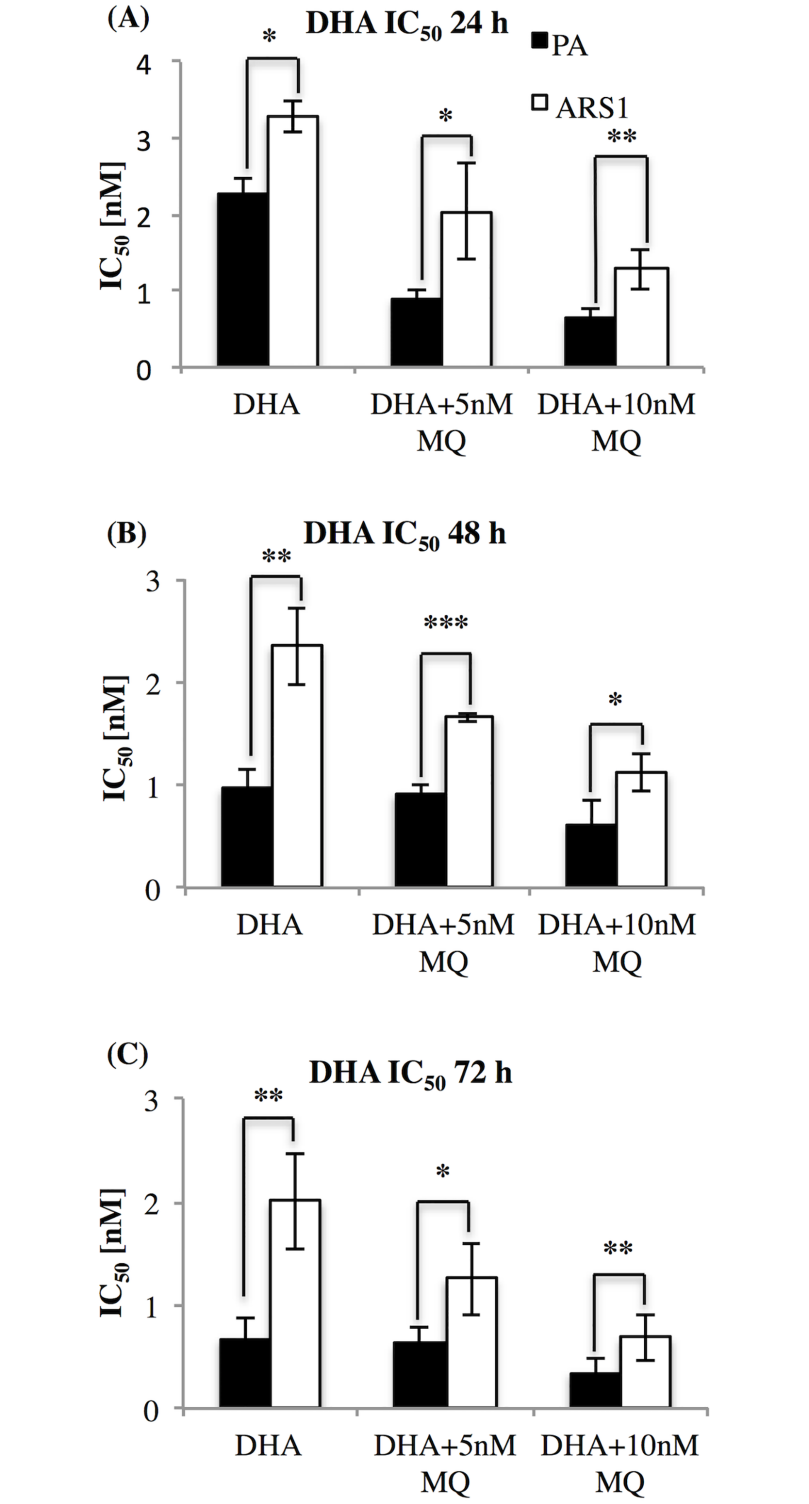
Statistical analysis of IC_50_ DHA in combination with MQ. PA and ARS1 were treated at rings stage (time 0) with 0.6, 1.25, 2.5, 5, 10 nM DHA, alone or in combination with 5 nM and 10 nM of MQ, and analysed at 24 h (A), 48 h (B) and 72 h (C). Data were normalized to the parasitemia measured at time 0 and the IC_50_ was calculated using ICEstimator software. Parasitemia was measured by microscopy of Giemsa-stained thin smears. Means +/- SD from 3 independent experiments is shown. Significant differences of parasitemia between strains are indicated by (*) at p < 0.05, (**) at p < 0.01 and (***) at p < 0.001.

The addition of MQ greatly decreased the IC_50_ against PA, signifying an increase in sensitivity to DHA while ARS1 sensitivity remained relative stable (Fig 5). The parasitemia was assessed and multiple time points to evaluate the antimalarial effect of DHA on the cultures and the response to long-acting MQ. ARS1 resistance appeared to be selective for a DHA. The DHA IC_50_ after 72 h correspond to 0.68 ± 0.2 nM and to 2 ± 0.1 nM for ARS1 while the IC_50_ of MQ alone after 72 h correspond to 5.49 ± 0.92 nM for PA and 4.78 ± 0.6 nM for ARS1.

Finally, mutations in the P*fk13* gene have been associated with ART resistance in *ex vivo* assays with P. *falciparum* strains obtained from the eastern Greater Mekong sub-region (Cambodia, Laos and Vietnam). The most common polymorphisms include C580Y, R539T, I543T and Y493H, so we sequenced the same regions in ARS1 and PA strains to help elucidate the underlying cause of this artemisinin phenotype. These regions were found to be identical in the two strains, eliminating this as possible causes of artemisinin resistance. Moreover, genotyping the *msp1*, *msp2 and glurp* genes, commonly used to differentiate reinfection from recrudescence in patients infected with malaria. This supports our findings that revealed no genetic diversity from these genes exists among PA and ARS1 and indicating that we have not selected a less represented strain that may have contaminated the parental strain.

The culmination of these results obtained form this study indicate that the ARS1 strain can be useful to better understand the mechanisms of ART resistance and for the evaluation of new antimalarial therapies in future studies.

## Discussion

Through a series of treatment with low concentrations of DHA, we selected for the P. *falciparum* artemisinin resistance strain (ARS1) that arose from the parental strain PA. The ARS1 resistant phenotype was maintained under monthly sub-lethal concentrations of DHA.

The study of Brauner et al. helps to define the phenotype observed in ARS1 as a state of “tolerance”, which is the capability of parasites to survive transient exposure to drugs, also without a consistent increase variation of IC_50_ [56].

Alarmingly, P. *falciparum* resistance to partner drugs along with ART in combination therapies has been detected, leaving populations in South East Asia vulnerable to deadly malaria infections [49, 28, 50]. For these reasons, a better understanding of the resistance mechanism will help the selection of more effective and resistance tolerant antimalarial therapies.

In the drug discovery pipeline, identifying target genes or molecules responsible for a drug, resistance phenotype is the first step in a 12–15 years process followed by high throughput screening for a candidate antimalarial compounds [57]. Despite artemisinin resistant isolates may more representative than ARS1, this could be an essential tool not only to elucidate the underlying mechanisms of artemisinin resistance but also to evaluate the efficacy of new compounds against these artemisinin resistant parasites.

To this end, we first evaluated the response of three artemisinin sensitive strains (PA, FCR3 and HB3A) to DHA, the active metabolite of all ART. Cultures were treated with low doses of DHA for 8 weeks to select for parasites demonstrating a drug tolerant phenotype. While many studies [25, 26] use high DHA concentrations (from 280 to 1200 nM) to generate resistant parasites, our method has the intended consequence of producing parasites that not enter a dormant or quiescent state, a state not demonstrated in patients. Our method intentionally prevents the accumulation of dormant parasites, using a maximum concentration of 5 nM DHA.

The artemisinin resistance phenotype of ARS1 is defined by the following features: i) adaptation to DHA is stable and can be maintained in drug-free medium for an extended period (at least 1 month); ii) a 3-fold increase in DHA IC_50_ values relative to the parental strain after 72 h (2 nM in ARS1 and 0.68 nM in PA); iii) a reduced susceptibility of ARS1 to repetitive pressure with low concentrations of DHA; iv) the ability to survive after a single pulse of 700 nM (PA: 0.51%, ARS1: 6.8%); v) resistance that appears specific to DHA, with no cross-resistance to other antimalarial drugs.

It should be, anyway, noted that, although ARS1 presents a consistent 3-fold increase of the DHA IC_50_ over its parental strain, the DHA IC_50_s of HB3A and ARS1 are similar, therefore, the IC_50_ should not be considered as an absolute index of ART resistance. On the contrary, ARS1 complies with the definition artemisinin resistant parasites, while no resistance was measurable in parental strain [32, 47]; as previously described by Witkowski, two strains with respectively ≤1% vs ≥1% of survival rate following 700 nM DHA treatment of survival rate, as PA and ARS1, are generally defined as sensitive and resistant strain.

Moreover, there was no presence of cross-resistance to other commonly used antimalarials and no increase in DHA sensitivity when combined with MQ (DHA-MQ). Despite we shouldn’t consider the IC_50_ value as an ART resistance index, in this case could be an important parameter to evaluate the tolerance of ARS1 to the MQ-DHA combination. It appears that ARS1 is selectively tolerant to DHA while maintaining its sensitivity to antimalarials present in ACTs [58, 59].

Although studies investigating the cause of artemisinin resistance are plentiful, the exact mechanism of resistance remains unclear. We postulated that a randomized molecular mutation caused the acquired resilience to DHA observed in ARS1 progeny. Genetic sequencing of the molecular markers *msp1*, *msp2* and *glurp* eliminated the possibility ARS1 acquired mutations in genes that are commonly used to discriminate parasite recrudescence from re-infection alleles in patients and was consistent with ARS1 arising from PA [53]. A mutation in the recently discovered P*fk13* propeller domain was also not found. A mutation in this domain would be consistent with other reports suggesting its association with artemisinin resistance in field isolates. The lack of P*fk13* propeller mutations observed in ARS1 cannot be interpreted due to the substantial lack of knowledge pertaining to the ART’ mechanism of action and to the mutation’s heterogeneity associated with delayed parasite clearance in different geographic populations [36]. However, it is of interest to further evaluate the cause of artemisinin resistance in this case and evaluate ARS1 for a ubiquitous source of the resistance phenotype.

In conclusion, the data obtained in the present study confirm the ability of P. *falciparum* cultures to become less responsive to DHA. We propose the ARS1 strain as a potential tool to study artemisinin resistance mechanisms and to test new antimalarial compounds.

## Supporting information

S1 Fig Representative images of P. *falciparum* cultures from the RSA^0-3h^. PA and ARS1 were treated with 700 nM of DHA. The survival of both cultures was monitored at time 0, 24 h, 72 h and 7 days following the addition of DHA. Parasites were monitored by Diff-Quick stained smears light microscopy using a 100x oil-immersion objective.
(TIFF)
