# Supplementary figures and images for: Induction of high tolerance to artemisinin by sub-lethal administration: A new in vitro model of P. falciparum
Source: PLoS One. 2018 Jan 17;13(1):e0191084. doi: 10.1371/journal.pone.0191084 (PMC5771598; doi:10.1371/journal.pone.0191084)

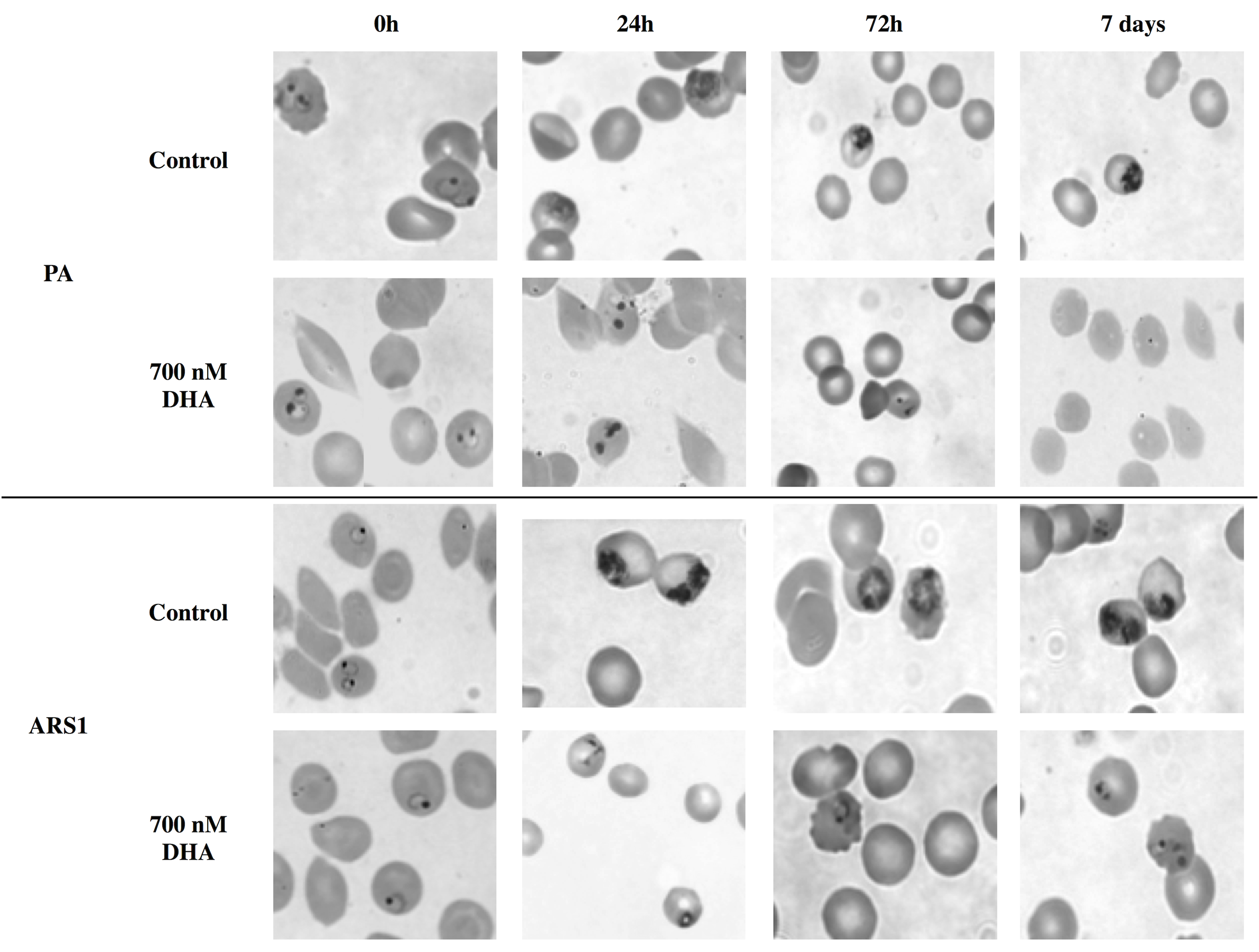

Supplement: S1 Fig — PA and ARS1 were treated with 700 nM of DHA. The survival of both cultures was monitored at time 0, 24 h, 72 h and 7 days following the addition of DHA. Parasites were monitored by Diff-Quick stained smears light microscopy using a 100x oil-immersion objective. (TIFF) [file pone.0191084.s001.tiff]
